# Lynch syndrome testing of colorectal cancer patients in a high-income country with universal healthcare: a retrospective study of current practice and gaps in seven australian hospitals

**DOI:** 10.1186/s13053-022-00225-1

**Published:** 2022-05-04

**Authors:** Julia Steinberg, Priscilla Chan, Emily Hogden, Gabriella Tiernan, April Morrow, Yoon-Jung Kang, Emily He, Rebecca Venchiarutti, Leanna Titterton, Lucien Sankey, Amy Pearn, Cassandra Nichols, Skye McKay, Anne Hayward, Natasha Egoroff, Alexander Engel, Peter Gibbs, Annabel Goodwin, Marion Harris, James G Kench, Nicholas Pachter, Bonny Parkinson, Peter Pockney, Abiramy Ragunathan, Courtney Smyth, Michael Solomon, Daniel Steffens, James Wei Tatt Toh, Marina Wallace, Karen Canfell, Anthony Gill, Finlay Macrae, Kathy Tucker, Natalie Taylor

**Affiliations:** 1 The Daffodil Centre, The University of Sydney, a joint venture with Cancer Council NSW, 153 Dowling St, NSW 2011 Woolloomooloo, Australia; 2 Surgical Outcomes Research Centre (SOuRCe), Royal Prince Alfred Hospital, Camperdown, NSW Australia; 3 NSW Health, Western Sydney Local Health District, Westmead, NSW Australia; 4 Monash Health, Melbourne, Victoria Australia; 5 Cancer Council NSW, Sydney, NSW Australia; 6 Genetic Services of Western Australia, King Edward Memorial Hospital, Perth, Western Australia Australia; 7 The Royal Melbourne Hospital, Melbourne, Victoria Australia; 8 School of Medicine and Public Health, University of Newcastle, Newcastle, NSW Australia; 9 The University of Sydney, Northern Clinical School Royal North Shore Hospital, Sydney, NSW Australia; 10 Personalised Oncology Division, Walter and Eliza Hall Institute, Melbourne, Victoria Australia; 11 Cancer Genetics Department, Royal Prince Alfred Hospital, Camperdown, NSW Australia; 12 Department of Tissue Pathology & Diagnostic Oncology, NSW Health Pathology, Royal Prince Alfred Hospital, Camperdown, NSW Australia; 13 Faculty of Medicine and Health, The University of Sydney, Sydney, NSW Australia; 14 School of Medicine and Pharmacology, University of Western Australia, Perth, Western Australia; 15 Centre for the Health Economy, Macquarie University, Sydney, NSW Australia; 16 Westmead Familial Cancer Services, The Crown Princess Mary Cancer Centre, Westmead Hospital, Westmead, NSW Australia; 17 Department of Colorectal Surgery, Royal Prince Alfred Hospital, Camperdown, NSW Australia; 18 Department of Colorectal Surgery, The University of Sydney, Westmead Hospital, Westmead, NSW Australia; 19 Fiona Stanley Hospital, South Metropolitan Health Service, Murdoch, Western Australia Australia; 20 Sydney Medical School, University of Sydney, Sydney, NSW Australia; 21 Colorectal Medicine and Genetics, Department of Medicine, The Royal Melbourne Hospital, Melbourne, Victoria Australia; 22 Hereditary Cancer Clinic, Prince of Wales Hospital, Sydney, NSW Australia; 23 Prince of Wales Clinical School, UNSW, Sydney, NSW Australia; 24 School of Population Health, Faculty of Medicine, UNSW, Sydney, NSW Australia

**Keywords:** Lynch syndrome, Mismatch repair, Tumour testing, Genetics services referral, Bottleneck, Gap, Heterogeneity in practice, Medical records

## Abstract

**Background:**

To inform effective genomic medicine strategies, it is important to examine current approaches and gaps in well-established applications. Lynch syndrome (LS) causes 3–5% of colorectal cancers (CRCs). While guidelines commonly recommend LS tumour testing of all CRC patients, implementation in health systems is known to be highly variable. To provide insights on the heterogeneity in practice and current bottlenecks in a high-income country with universal healthcare, we characterise the approaches and gaps in LS testing and referral in seven Australian hospitals across three states.

**Methods:**

We obtained surgery, pathology, and genetics services data for 1,624 patients who underwent CRC resections from 01/01/2017 to 31/12/2018 in the included hospitals.

**Results:**

Tumour testing approaches differed between hospitals, with 0–19% of patients missing mismatch repair deficiency test results (total 211/1,624 patients). Tumour tests to exclude somatic MLH1 loss were incomplete at five hospitals (42/187 patients). Of 74 patients with tumour tests completed appropriately and indicating high risk of LS, 36 (49%) were missing a record of referral to genetics services for diagnostic testing, with higher missingness for older patients (0% of patients aged ≤ 40 years, 76% of patients aged > 70 years). Of 38 patients with high-risk tumour test results and genetics services referral, diagnostic testing was carried out for 25 (89%) and identified a LS pathogenic/likely pathogenic variant for 11 patients (44% of 25; 0.7% of 1,624 patients).

**Conclusions:**

Given the LS testing and referral gaps, further work is needed to identify strategies for successful integration of LS testing into clinical care, and provide a model for hereditary cancers and broader genomic medicine. Standardised reporting may help clinicians interpret tumour test results and initiate further actions.

**Supplementary information:**

The online version contains supplementary material available at 10.1186/s13053-022-00225-1.

## Background

Colorectal cancer (CRC) is one of the most common cancers, with ~ 1.9 million new diagnoses and > 935,000 deaths globally in 2020 [1]. Approximately 3–5% of CRCs are due to Lynch syndrome (LS) [2], an inherited predisposition to cancer (previously called “hereditary non-polyposis colorectal cancer”). Early detection of LS is key, as it gives individuals access to cancer risk management strategies including colonoscopic surveillance, which reduces CRC mortality [3]. Moreover, it allows for cascade testing of relatives and risk management for those who also have LS [4].

Guidelines in many countries including the US, UK and Australia recommend screening of all CRC patients for LS, generally using a step-wise process (see Additional Files 1 and 2) [4–9]. The availability of long-standing recommendations and cost-effective, evidence-based testing strategies make LS an important example for genomic medicine [10].

LS is mostly due to pathogenic germline genetic variants in DNA mismatch repair genes (*MLH1*, *MSH2*, *MSH6*, *PMS2*), or a deletion of the *EPCAM* gene causing epigenetic *MSH2* silencing [7]. Deficient mismatch repair (dMMR) often leads to microsatellite instability (MSI) [7]; however, MSI/dMMR also occurs in ~ 15% of non-LS colorectal tumours due to somatic hypermethylation of the *MHL1* promoter [11].

Individuals with high risk of LS based on tumour tests (here and below, dMMR/MSI, plus *BRAF* V600E and/or *MLH1* promoter hypermethylation tests where needed) should be referred for genetic counselling and germline genetic testing as appropriate. Tumour testing practice varies between contexts (see Additional File 1); in some settings, germline genetic testing for LS can also be done directly by surgeons/oncologists (e.g. in Australia since 2020) [12].

A key aim of the testing and referral guidelines is to improve health outcomes for LS carriers and their relatives. Missed opportunities to identify LS carriers through gaps in tumour testing or referrals for genetic counselling and germline genetic testing where appropriate lead to missed opportunities in prevention and early detection of potential metachronous cancers for these individuals, with a subsequent burden of more aggressive treatment or even cancer deaths that could have been prevented. Similarly, missed opportunities to identify LS carriers among CRC cancer patients also leads to missed opportunities for cascade testing of relatives, with analogous potential of late cancer diagnoses and deaths that could have been prevented.

Australia is a high-income country with universal healthcare aiming to move towards routine genomic medicine in practice [13]. Knowledge of current LS testing practice in Australia can therefore provide insights on gaps that can occur even in high-income countries and highlight key areas to address for future genomic medicine approaches. To the best of our knowledge, only four studies have assessed LS tumour testing practice in the Australian healthcare context [14–17], and only included 1–2 hospitals or genetics services in one state during different study periods (see Additional File 1), limiting insights on the heterogeneity of practice. Moreover, only one [17] of these studies included more recent data up to 2017.

In this study, we characterise the tumour testing and referral approaches in seven large hospitals located in three different states in Australia in 2017–2018, highlighting key gaps and variation in practice.

## Methods

### Study population

This study was carried out in conjunction with an implementation trial to improve LS testing [18]. Patients with CRC resection in the two calendar years prior to the start of the trial were included, ensuring none of the tumour testing and referral rates would be influenced by the trial itself. Specifically, this study included seven public hospitals (H1-H7 in the following) from three states in Australia: New South Wales (four hospitals), Victoria (two hospitals), and Western Australia (one hospital). Ethics approval for this study was granted by the Royal Prince Alfred Hospital Human Research Ethics Committee (reference HREC/17/RPAH/542); the ethics committee approved a waiver of consent from individual patients to collect the clinical data. Site-specific governance approval was obtained from each hospital site.

In each hospital, we identified all patients who underwent a CRC resection from 01/01/2017 to 31/12/2018. Depending on the hospital system, patients were identified from electronic hospital databases or surgeons’ records (see Additional File 3 for details). For each patient, pathology data were obtained from electronic patient medical records or using linkage to a pathology database. Throughout this study, data were obtained using structured queries of electronic databases where possible, supplemented with manual checks and additional data extraction (see Additional File 3).

We excluded patients with neuroendocrine, nerve sheath, granular cell, yolk-sac, stromal or appendiceal mucinuous tumours, pseudomyxoma peritonei, squamous cell carcinoma, goblet cell carcinoid, acellular mucin (H1: *n* = 8; H2: *n* = 6; H2: *n* = 0; H7: *n* = 1; H4: *n* = 16; H3: *n* = 0; H6: *n* = 3). While for H3, tumour type could only be checked for patients without immunohistochemistry test results (see below), all of these were adenocarcinoma (so that tumour type would not cause missing test results). To ensure that tumour testing was possible, patients with a medical record note of no/low tumour tissue at resection were excluded (H1: *n* = 23; H2: *n* = 18; H2: *n* = 22; H7: *n* = 5; H4: *n* = 21; H3: *n* = 8; H6: *n* = 11). Finally, we excluded patients with known familial adenomatous polyposis (n < 5; exact number suppressed to preserve confidentiality).

### Data on tumour testing and referral to genetics services

Reports for the following tests were obtained using pathology data and patient medical records as above: immunohistochemistry (IHC) tests for dMMR (i.e. loss of at least one MMR protein), PCR tests for MSI, *BRAF* V600E tests, and *MLH1* promoter hypermethylation tests. Where no report for testing of the tumour sample was available, electronic medical records for tests undertaken on biopsy samples were checked manually. Data on discussion of patients at multidisciplinary team (MDT) meetings was available for all hospitals except H2.

To identify patients referred to genetics services, we obtained a list of CRC patients from the genetics service (or familial cancer centre, hereafter included in “genetics services”) working with each of the hospitals, linking data based on patient ID, name, and date of birth or surgery (see Additional File 3). This included records for patients referred to the genetics service by a clinician, general practitioner (GP) or who self-referred; we separately noted where patients’ hospital records stated referral to a different genetics service. For each patient included in the study, presence or absence of a genetics service referral record, attendance of the patient at the genetics service, and results of any relevant diagnostic genetic tests were noted, including available records of these events that occurred prior to the CRC resection (to allow for e.g. prior referral based on family history or cascade testing). To allow for a lag between the resection and referral, genetics service data were extracted to 28/02/2019.

After patients’ resection, pathology, and genetics service data were linked at each hospital, all patient information was de-identified and securely transmitted to the central study team for analysis.

### Data analysis

All analyses were done in R v3.6.0.

We calculated the number and percentage of patients with specific tumour test results: (1) both dMMR and MSI test missing; (2) dMMR/MSI detected; (3) MLH1 loss; (4) MLH1 loss with no *BRAF* V600E nor *MLH1* promoter hypermethylation test; (5) MLH1 loss with *BRAF* V600E or *MLH1* promoter hypermethylation detected.

For H1, testing guidelines changed in late 2017, so the number of patients with dMMR/MSI tests missing was also quantified separately based on 2017 and 2018 data.

We calculated the number of patients with tumour testing completed and the results indicating high risk of LS (i.e. at least one of: 1) high MSI, 2) MSH2 loss, 3) MSH6 loss, 4) PMS2 loss but no MLH1 loss, or 5) MLH1 loss with no *BRAF* V600E nor *MLH1* promoter hypermethylation detected), and the proportion of these patients with a record of referral to genetics services (including any available records of referrals before or after the CRC resection). “High LS risk” here does not include patients with tumour MLH1 loss but a *BRAF* V600E variant or *MLH1* promoter hypermethylation detected, as they were not generally considered to require a genetics services referral.

We assessed pairwise differences between hospitals for three gaps in testing and referral (Fisher’s test): missing (1) both dMMR and MSI test results; (2) *BRAF* V600E and *MLH1* promoter methylation test results with tumour MLH1 loss; (3) record of referral to genetics services for patients with all tumour tests complete and indicating high LS risk. We also compared the number of patients with dMMR/MSI between hospitals (Fisher’s test). To account for multiple testing, significance was defined as *p* < 0.0024 (Bonferroni correction for 21 pairwise comparisons).

We assessed differences between the ages of patients who had tumours with versus without dMMR/MSI (two-sample Wilcoxon test); in a *post hoc* analysis, this test was repeated for patients aged < 70 years.

For patients who had all tumour tests complete and results indicating high LS risk (see above), we also tested for (1) differences between ages of those who did versus did not have a record of referral to genetics services (two-sample Wilcoxon test); (2) association between a record of referral and discussion of patients at MDT meetings (Fisher’s test); (3) association between a record of referral to genetics services and age, sex, MDT discussion, and hospital in a joint model (logistic regression, complete case analysis).

Finally, for patients with all tumour tests complete and indicating high LS risk who had a diagnostic genetic test, we tested for differences in age between those with and without a relevant pathogenic/likely pathogenic variant (two-sample Wilcoxon test).

## Results

We included 1,624 patients who underwent a CRC resection from 01/01/2017 to 31/12/2018, with 116–382 patients per hospital (Table 1). The majority of patients were male (57.1%), and 28.9% were aged ≤ 60 years at resection.

**Table 1:** Characteristics of colorectal cancer patients included in the study

Hospital	Number of patients	Number of female patients (%)	Mean age of patients (range)	Number of patients aged ≤ 60 years (%)
H1	271	114 (42.7%)	67.9 (30–92)	74 (27.3%)
H2	382	171 (44.8%)	67.6 (26–94)	106 (27.7%)
H3	123	51 (41.5%)	66.5 (30–90)	40 (32.5%)
H4	311	127 (40.8%)	64.5 (22–94)	109 (35.0%)
H5	251	102 (40.6%)	69.1 (29–94)	61 (24.3%)
H6	170	81 (47.6%)	67.6 (32–92)	44 (25.9%)
H7	116	51 (44.0%)	67.9 (26–95)	35 (30.2%)
Total	1,624	697 (42.9%)	67.2 (22–95)	469 (28.9%)

**Fig. 1:**
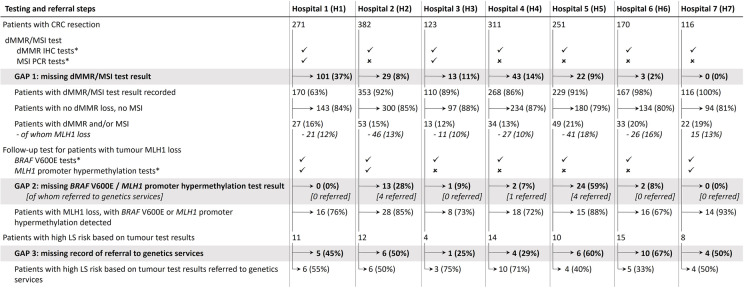
LS tumour testing and referral to genetics services at seven Australian hospitals in 2017–2018. The percentages in each box are calculated relative to the number of patients in the previous testing step. The numbers of patients with (i) MSH2 or MSH6 loss, or (ii) PMS2 loss only, together account for < 10 patients per hospital and are not shown separately to protect patient confidentiality. * test used for at least some patients

The seven hospitals had different approaches to LS tumour testing (Fig. 1): five hospitals used dMMR IHC tests, while two (H1, H3) also used MSI PCR tests for some patients. For patients aged 60 + years at H1, dMMR/MSI testing was only undertaken upon explicit clinician request in 2017, with testing of all patients even without explicit requests (“universal testing”) introduced in 2018. All other hospitals introduced universal testing before 2017. To check whether MLH1 loss (where present) is likely somatic only, four hospitals used *BRAF* V600E tests only, while three also used *MLH1* promoter hypermethylation tests.

Overall, we detected pervasive tumour testing gaps in the majority of hospitals. dMMR/MSI test results were present for all patients in only one of seven hospitals (H7), closely followed by another hospital (H6; missing results for 2% of patients). Testing gaps were more substantial at other hospitals: dMMR/MSI test results were missing for 8–14% of patients at H2-H5, and 37% of patients at H1 (2017: 8% and 79% of patients aged < 60 and 60 + years, respectively; 2018: 19% of patients). In total, 13% (211/1624) patients had no dMMR/MSI test result recorded. For these and other missing tests described below, p-values for differences between hospitals are shown in Additional File 4, with generally significant differences between the hospitals with the highest and lowest missingness rates.

**Fig. 2:**
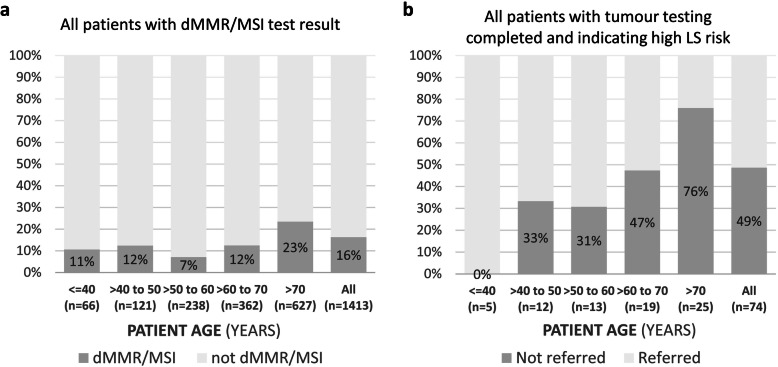
dMMR/MSI test results and referral to genetics services by patient age group. **a** dMMR/MSI tumours were more common in older patients (Wilcoxon *p* = 3.7 × 10^− 13^ for difference in ages of patients with and without dMMR/MSI). **b** Older patients with tumour test results complete and indicating high LS risk were less likely to be referred to genetics services (Wilcoxon *p* = 9.8 × 10^− 5^ for difference in ages of patients with and without referral record)

Of the 1,413 patients with a recorded dMMR/MSI test result, 16% had dMMR tumours (13% MLH1 and PMS2 loss, 1% MSH2 and MSH6 loss, 1% PMS2 loss only, < 1% MSH6 loss only, none MSI only). Overall, patients with tumour dMMR were older (Fig. 2 A; Wilcoxon *p* = 3.7 × 10^− 13^). However, a *post-hoc* analysis showed this was mainly due to higher dMMR rates in patients aged 70 + years (Wilcoxon *p* = 0.75 when restricting the analysis to patients aged < 70 years).

In total, 22% (42/187) of patients with tumour MLH1 loss had missing results for both *BRAF* V600E and *MLH1* promoter hypermethylation tests. *BRAF* V600E test results were recorded for all patients with tumour MLH1 loss at two hospitals (H1, H7), of which one (H7) further included *MLH1* promoter hypermethylation test results where no somatic *BRAF* V600E variant was found. At the other five hospitals, the follow-up testing was missing for 7–59% of patients with MLH1 loss (Fig. 1; Additional File 4). Where present, the follow-up testing detected likely somatic *MLH1* inactivation in 79% (115/145) of patients with MLH1 loss, underscoring the importance of this test to identify patients with low LS risk.

Of all patients with full tumour testing completed (dMMR/MSI and where needed, a *BRAF* V600E or *MLH1* hypermethylation test), a result indicating high LS risk was present in 5% (74/1371). However, we could not find evidence of a referral to genetics services for 49% (36/74) of these patients. Lack of referral was strongly associated with older age (Fig. 2B; none missing in patients aged ≤ 40 years, one in three missing for patients aged > 40 to 60 years, one in two missing for patients aged > 60 to 70 years, and three in four missing for patients aged > 70 years), but not with discussion of patients at multidisciplinary team meetings (see Additional File 1). Of the 38 patients with a referral record, records of genetics services consultations were available for 28 (74%), 25 (89% of 28) had a diagnostic genetic test, and 11 (44% of 25) had a pathogenic/likely pathogenic variant in *MLH1/PMS2*/*MSH2*/*MSH6*, thus qualifying for a LS diagnosis (see Additional Files 1 and 5).

## Discussion

In summary, we have analysed LS tumour testing and referral to genetics services for 1,624 CRC patients in seven Australian hospitals in 2017–2018 (24 months). We found three areas with pervasive gaps: missing dMMR/MSI test records at five of seven hospitals (overall ~ 1 in 10 patients); missing follow-up testing of patients with MLH1 loss at five hospitals (overall ~ 1 in 5 of patients with MLH1 loss); missing record of referral to genetics services for ~ 1 in 2 patients with tumour testing complete and indicating high LS risk.

In view of the increasing interest in integrating genomic technologies into clinical practice [18–20], the identification of wide-spread gaps in the context of well-established LS testing shows that additional research is needed to identify best-practice approaches to genomic testing and determine how to support their implementation. At the seven hospitals in this study, a clinical trial to design interventions addressing the above gaps in LS testing and referral is currently underway [18]. This includes the identification of barriers to appropriate testing and referral as perceived by a wide range of different stakeholders at each hospital, which may also be informative for other applications of genomic medicine. In particular, there are likely mutliple psychosocial and contextual factors contributing to tumour testing and referral gaps, such as mixed perceptions of healthcare staff around the utility of tumour testing and referral, high workload and competing priorities, lack of clarity around processes and roles, difficulty interpreting tumour test results and challenges around remembering criteria for referral to genetics services. Ultimately, the clinical trial aims to test which interventions could support healthcare staff for relevant steps of the tumour testing and referral pathway.

As an example of excellent practice, LS tumour testing of all CRC patients with highly standardised processes was demonstrated by one hospital in this study (H7). While extracting the exact wording of all pathology reports and whether they used synoptic reporting was outside the scope of this work, we obtained some examples of pathology reports (see Additional File 6). These suggest the reports for H7 were highly standardised, with more variability in phrasing at some other hospitals, especially for results indicating high LS risk.

As suggested by the Australian Gastrointestinal Pathology Society [8], standardised reporting might help clinicians interpret test results and initiate further actions. Electronic medical records with standardised forms, easy access to reliable knowledge, and clinical decision support systems may also assist effective integration of genomics into clinical workflows [21]. However, appropriate referral and germline genetic testing also involves obtaining informed consent from patients, requiring both genomics knowledge and communication/counselling skills.

A previous study in the USA also suggested that the involvement of genetics services earlier in the testing and referral pathway could substantially improve referral rates: in that context, referral to genetics services was both faster and more comprehensive during a period when tumour test results were immediately sent to both colorectal surgeons and genetic counsellors, and the genetic counsellors contacted patients directly [22]. By contrast, when only surgeons received the tumour test results, about 1 in 2 CRC patients with tumour dMMR or MSI in that hospital were not referred to genetics services, similar to the average rate observed in our study. In alignment with past reviews [23] and the Australian Medical Association 2020 statement on Genetic Testing and Genomics in Medicine [24], this also suggests that training of clinicians is key to successful integration of genomic testing into clinical practice. One would expect such training to play an even more important role in the future, with, for example, recent changes enabling surgeons or oncologists in Australia to directly order germline LS genetic testing without prior referral to genetics services.

While we did not observe an association between a record of referral to genetics services and discussion of patients with molecular results indicating high risk of LS at an MDT meeting, this could be due to limited sample size. For most hospitals, we were also not able to obtain the dates of MDT meetings and thus could not determine whether the tumour test results were available for that discussion. For example, for hospital H1, the complete tumour test results were available at the time of the MDT discussion for 3/5 patients with high-risk results who were referred to genetics services, but only for 1/5 patients without a record of referral. Consequently, further data are needed to determine whether and how systematic discussion of patients with high-risk tumour test results at MDT meetings could help improve risk-appropriate referrals to genetics services.

A particular challenge for identifying and applying best practice is the availability and accessibility of up-to-date clinical data (detailed discussion see Additional File 1). The results presented here are based on data for 2017–2018, noting that pathology practice since then may have changed and impacted the extent of testing gaps. International guidelines to recommend tumour testing of CRC patients have emerged since 2009 [4, 5, 7], and universal testing was thought to be in place for six of the seven hospitals in this study by 2017 (and in all seven by 2018). However, testing practice could have been further improved by the Australasian Gastrointestinal Pathology Society consensus practice guidelines [8], which endorsed universal testing of all CRC patients. Thus, it will be important to continue examining LS tumour testing and referral practice in Australia beyond 2018. In clinical practice, improved data management and research governance could facilitate internal hospital audits and encourage learning health systems with better integration of research and clinical practice [25]. Meanwhile, research data as presented here can help showcase best-practice achievements.

A limitation of our study is that patients with metastatic cancer and no CRC resection were not included. While we employed a consensus approach to ensure between-hospital data comparability (see Methods, Additional Files 3, 7 and 8), some information for patients undergoing CRC resections may have been missed due to data extraction errors or incomplete records. To mitigate this, data extraction was carried out by healthservice professionals employed within the healthcare system, extensively consulting hospital stakeholders to optimise data access and coverage, and combining information from multiple systems where necessary. Finally, we could not identify where referral to genetics services was declined by a patient or not possible due to their ill-health/death (more likely among older patients, for whom referral records were less common). While these factors could preclude some referrals, missed LS diagnoses also reduce prevention and surveillance opportunities for patients’ relatives, which was identified as particularly important by a patient representative who was part of the study team.

Our work also has notable strengths: inclusion of multiple hospitals in different Australian states, use of clinical data and electronic health records where available, and identification of referrals using data from genetics services.

## Conclusions

Tumour testing for LS is widely adopted at hospitals in Australia, but we have found that gaps in practice remain at many hospitals in different states. Further work is needed to identify the procedures, funding structures, and targeted implementation strategies that can ensure successful implementation of universal testing in the LS context, and inform approaches for future broader integration of genomic testing pathways into clinical practice.

## Supplementary information

**Additional file 1:** Supplementary Notes.

**Additional file 2:** Conceptual map of a step-wise tumour testing approach for LS.

**Additional file 3:** Supplementary Methods.

**Additional file 4:** *P*-values for pairwise differences for missing test results and referrals to genetics services between hospitals.

**Additional file 5:** Diagnostic genetic testing among patients with complete, high-risk tumour test results who had a referral to genetic services.

**Additional file 6:** Examples of different terminology for LS tumour test results used in pathology reports.

**Additional file 7:** Consensus list of Medicare Benefit Schedule (MBS) procedure codes used to identify colorectal resections (in conjunction with ICD codes for CRC diagnosis).

**Additional file 8:** Australian Classification of Health Interventions (ACHI) procedure codes mapped to consensus list of MBS items as an alternative to identify colorectal resections (in conjunction with ICD codes for CRC diagnosis).

## Data Availability

Individual-level patient data are not publicly available to preserve patients’ privacy. Reasonable requests for data can be submitted to the corresponding author and will be subject to ethics committee and individual hospital governance requirements.

## Abbreviations

LS: Lynch syndrome
CRC: Colorectal cancer
dMMR: Deficient mismatch repair
MMR: Mismatch repair
MSI: Microsatellite instability
IHC: Immunohistochemistry
PCR: Polymerase chain reaction
MDT: Multidisciplinary team
GP: General practicioner
ICD: International Classification of Diseases
MBS: Medicare Benefit Schedule
ACHI: Australian Classification of Health Interventions
