# Supplementary material for: Lynch syndrome testing of colorectal cancer patients in a high-income country with universal healthcare: a retrospective study of current practice and gaps in seven australian hospitals
Source: Hered Cancer Clin Pract. 2022 May 4;20:18. doi: 10.1186/s13053-022-00225-1 (PMC9066828; doi:10.1186/s13053-022-00225-1)
Supplement: Supplementary file 1 — Additional file 1: Supplementary Notes. [file 13053_2022_225_MOESM1_ESM.docx]

**Lynch syndrome testing of colorectal cancer patients in a high-income country with universal healthcare: a retrospective study of current practice and gaps in seven Australian hospitals**

**SUPPLEMENTARY NOTES**

[**CRC tumour testing** 1](#_Toc92897767)

[**Referral to genetics services** 2](#_Toc92897768)

[**Diagnostic genetic testing** 2](#_Toc92897769)

[**Availability and accessibility of up-to-date clinical data** 3](#_Toc92897770)

[**Limitations** 3](#_Toc92897771)

[**Supplementary References** 3](#_Toc92897772)

#### **CRC tumour testing**

Typically, a CRC tumour sample is tested for dMMR via immunohistochemistry (IHC) of the four MMR proteins, or for MSI using a polymerase chain reaction test (Additional File 2). IHC testing is often preferred as it is cheaper and helps direct and interpret subsequent germline genetic testing for specific genes. For tumours with loss of MLH1 or high MSI, a second testing step can be undertaken to exclude somatic inactivation with low likelihood of LS (Additional File 2), indicated by *MLH1* promoter hypermethylation, or, alternatively, the presence of a somatic *BRAF* V600E variant. The *BRAF* V600E variant is very rare in individuals with LS and present in ~70% of cases with *MLH1* promoter hypermethylation(1) (overall estimated negative predictive value ~40%(2), but <15% for individuals aged 70+ years; possibly even lower in a non-Western population(3)). Consequently, some recommendations suggest negative *BRAF* V600E tests be followed by an *MLH1* promoter hypermethylation test(2). Individuals with high risk of LS based on tumour tests should be referred for genetic counselling and germline genetic testing as appropriate. In some settings, germline genetic testing for LS can also be ordered directly by surgeons or oncologists, without referral to tertiary genetics services (e.g. introduced in Australia in 2020(4)). Compared to germline genetic testing of all CRC patients, the step-wise approach can be more cost-effective(5).

A systematic review published in 2017(6) identified five studies on tumour testing for LS. The included studies covered five different settings (Ohio(7), California(8), Washington(9) in the USA; Switzerland(10); Western Australia(11)) and different periods in 2008-2015. Since 2017, additional studies have been published using data from hospitals (e.g. Ireland(12), Australian Capital Territory(13)) or research cohorts (e.g. in Australia(14)). These studies cover a wide range of contexts, with findings illustrating both complete tumour testing of all CRC patients in some settings(10), and testing gaps in some of the included hospitals in other settings (e.g. 0%, 56% and 84% of patients missing dMMR/MSI test results at three centres in Ireland in 2005-2013(12)).

In Australia, to the best of our knowledge, only four studies have assessed LS tumour testing practice in the healthcare context(11, 13, 15, 16). The earliest (included in the systematic review described above) considered CRC patients in Western Austalia in 1994-2012(11); IHC results were used as an inclusion criterion and was based on patients meeting one of three criteria: 1) patients aged <60 years, (2) patients with an individual or family history of cancer, or (3) patients whose tumours have histological characteristics suggestive of LS. A study of a hospital in the Australian Capital Territory included 1,612 CRC patients with IHC tests in 2004-2013, with *BRAF* V600E or *MLH1* promoter hypermethylation tests only carried out after referral to genetics services during that period.(13) A study of two NSW hospitals in 2015-2016 (n=350 patients total) also found gaps in referral to genetic counselling and testing (20-90% and 45-90% of patients not referred before and after implementation of interventions)(15). Finally, a study of 1,171 CRC patients in a hospital in Victoria in 2010-2017 found that IHC for dMMR was only performed for 680 patients during that period, with testing for patients 59-60 and >60 years of age becoming more common in later years (e.g. <20% of patients aged >60 years tested in 2011-2012, but >90% tested in 2017). Of 88 with abnormal IHC results (excluding those with MLH1 loss and a *BRAF* V600E mutation, but including those with a missing *BRAF* V600E test), only 44 were referred to genetics services (50%), similar to the referral rates in the current study. 29 of these 44 patients attended genetics services (66%), 16 underwent diagnostic genetic testing for LS (55% of 29), leading to a LS diagnosis for 7 patients (44% of 16). Of the 44 patients with dMMR tumour test who were not referred to genetics services, 91% were over 60 years of age, in agreement with the findings of our study that missing records of referral to genetics services are strongly associated with increased age.

The gaps in tumour testing identified here and the heterogeneity between hospitals agrees with the findings of previous Australian and international studies, e.g. a study of three centres in Ireland in 2005-2013(12), and a study of two Australian hospitals in 2014-2016(15). Notably, supporting the quality of data collected in our work, the proportion of patients whose tumour demonstrated dMMR or MSI (average 16%) was in line with estimates from the Australian and international studies included in the recent systematic review(6) (e.g. 16%(7), 19%(8), 15%(10), 11.1-12.5%(14), 10-20%(12) and 17%(13)).

#### **Referral to genetics services**

There was a non-significant trend for higher referral of patients discussed at multidisciplinary team (MDT) meetings (among patients with high-risk tumour test results excluding H2, referral records were present for 25/43 (58%) patients discussed and 7/19 (37%) patients not discussed at an MDT meeting, odds ratio 2.35, p=0.17). A multivariate analysis confirmed significant association of referral to genetics services with age (adjusted odds ratio 0.94 per increased year of age, 95% confidence interval 0.89-0.98, p=0.0048), but not hospital or sex, with further attenuated association with MDT discussion (adjusted odds ratio 1.76, p=0.55).

#### **Diagnostic genetic testing**

Of the 38 patients with full tumour testing completed and indicating high risk of LS who had a record of referral to genetics services, 74% (n=28) had a record of attending a genetics services appointment (noting that 2 patients were referred to other genetics services for which attendance records were unavailable, Additional File 5). Of these, 25 patients had a diagnostic genetic test, and 11 (44% of 25) were identified to have a pathogenic or likely pathogenic variant in MLH1/PMS2/MSH2/MSH6, thus qualifying for a LS diagnosis. This included some patients for whom the referral and/or testing was carried out prior to resection (e.g. due to younger age, a previous cancer diagnosis, or family history). Patients with a relevant pathogenic or likely pathogenic variant were on average younger compared to patients for whom genetic testing did not identify such a variant (median age 48 versus 59 years, 25-75% range 38-55 years versus 51-69 years, Wilcoxon test p=0.034).

#### **Availability and accessibility of up-to-date clinical data**

A particular challenge impeding the identification and application of best practice is the availability and accessibility of up-to-date clinical data. In our study, 33 months passed between the initiation of the work and successful governance approval in all sites, and a further two months until completion of data extraction at all sites. This created a gap between the practice covered by the data (2017-2018) and the practice in place by the time the data were analysed and published. From a research perspective, this further complicates already complex implementation trials aiming to study the system in real time as improvement approaches are attempted. From a clinical perspective, improved data management systems and research governance processes could facilitate internal audits within hospitals and encourage learning health systems with better integration of research and clinical practice(17). These aspects could also facilitate collaboration between hospitals, helping place the results of internal audits in the context of practice elsewhere, and making it easier to identify and learn from “gold-standard” approaches in real time. However, we also acknowledge that safeguarding patient privacy is a key concern to ensure and preserve trust in healthcare, hence the development of an improved system poses some major challenges. In the meantime, research data as presented here can help showcase best-practice achievements.

#### **Limitations**

Further to the limitations described in the main text, some patient data held at the hospital might have been missed, e.g. additional tests or referrals actioned after the data was extracted, or tests carried out on biopsy samples at a different hospital. However, we note that even if biopsy tissue was tested elsewhere, by the current hospital procedures, the tests of tumour tissue from resection would still have been warranted.

#### **Supplementary References**

1. Palomaki GE, McClain MR, Melillo S, Hampel HL, Thibodeau SN. EGAPP supplementary evidence review: DNA testing strategies aimed at reducing morbidity and mortality from Lynch syndrome. Genet Med. 2009;11(1):42-65.

2. Adar T, Rodgers LH, Shannon KM, Yoshida M, Ma T, Mattia A, et al. A tailored approach to BRAF and MLH1 methylation testing in a universal screening program for Lynch syndrome. Mod Pathol. 2017;30(3):440-7.

3. Wang W, Dong L, Zou S, Lu N. Use of MLH1 methylation analysis versus BRAF V600E mutation testing to select patients for Lynch syndrome genetic testing in a Chinese population: A large consecutive cohort study. Journal of Clinical Oncology. 2019;37(4_suppl):493-.

4. Australian Government Department of Health. Medicare Benefits Schedule - Item 73354 2020 [Available from: http://www9.health.gov.au/mbs/fullDisplay.cfm?type=item&q=73354&qt=item.

5. Kang YJ, Killen J, Caruana M, Simms K, Taylor N, Frayling IM, et al. The predicted impact and cost-effectiveness of systematic testing of people with incident colorectal cancer for Lynch syndrome. Med J Aust. 2020;212(2):72-81.

6. Tognetto A, Michelazzo MB, Calabró GE, Unim B, Di Marco M, Ricciardi W, et al. A Systematic Review on the Existing Screening Pathways for Lynch Syndrome Identification. Front Public Health. 2017;5:243.

7. Heald B, Plesec T, Liu X, Pai R, Patil D, Moline J, et al. Implementation of universal microsatellite instability and immunohistochemistry screening for diagnosing lynch syndrome in a large academic medical center. J Clin Oncol. 2013;31(10):1336-40.

8. Kidambi TD, Lee R, Terdiman JP, Day L. Successful implementation of Lynch syndrome screening in a safety net institution. J Community Genet. 2016;7(3):255-60.

9. Cohen SA, Laurino M, Bowen DJ, Upton MP, Pritchard C, Hisama F, et al. Initiation of universal tumor screening for Lynch syndrome in colorectal cancer patients as a model for the implementation of genetic information into clinical oncology practice. Cancer. 2016;122(3):393-401.

10. Zumstein V, Vinzens F, Zettl A, Heinimann K, Koeberle D, von Flüe M, et al. Systematic immunohistochemical screening for Lynch syndrome in colorectal cancer: a single centre experience of 486 patients. Swiss Med Wkly. 2016;146:w14315.

11. Schofield L, Grieu F, Amanuel B, Carrello A, Spagnolo D, Kiraly C, et al. Population-based screening for Lynch syndrome in Western Australia. Int J Cancer. 2014;135(5):1085-91.

12. O'Kane GM, Ryan É, McVeigh TP, Creavin B, Hyland JM, O'Donoghue DP, et al. Screening for mismatch repair deficiency in colorectal cancer: data from three academic medical centers. Cancer Med. 2017;6(6):1465-72.

13. Brennan B, Hemmings CT, Clark I, Yip D, Fadia M, Taupin DR. Universal molecular screening does not effectively detect Lynch syndrome in clinical practice. Therap Adv Gastroenterol. 2017;10(4):361-71.

14. Buchanan DD, Clendenning M, Rosty C, Eriksen SV, Walsh MD, Walters RJ, et al. Tumor testing to identify lynch syndrome in two Australian colorectal cancer cohorts. J Gastroenterol Hepatol. 2017;32(2):427-38.

15. Long JC, Debono D, Williams R, Salisbury E, O'Neill S, Eykman E, et al. Using behaviour change and implementation science to address low referral rates in oncology. BMC Health Serv Res. 2018;18(1):904.

16. Loh Z, Williams DS, Salmon L, Dow E, John T. Impact of universal immunohistochemistry on Lynch syndrome diagnosis in an Australian colorectal cancer cohort. Intern Med J. 2019;49(10):1278-84.

17. Clay-Williams R, Taylor N, Braithwaite J. Potential solutions to improve the governance of multicentre health services research. Medical Journal of Australia. 2018;208(4):152-4.
