# Supplementary material for: Lynch syndrome testing of colorectal cancer patients in a high-income country with universal healthcare: a retrospective study of current practice and gaps in seven australian hospitals
Source: Hered Cancer Clin Pract. 2022 May 4;20:18. doi: 10.1186/s13053-022-00225-1 (PMC9066828; doi:10.1186/s13053-022-00225-1)

**Lynch syndrome testing of colorectal cancer patients in a high-income country with universal healthcare: a retrospective study of current practice and gaps in seven Australian hospitals**

**Conceptual map of a step-wise tumour testing approach for LS.**

Other approaches (e.g. immediate genetic test ordered by oncologist or surgeon) are also possible, depending on the relevant clinical guidelines. Depending on the setting, tumour testing can also be restricted to colorectal cancer patients with specific characteristics (e.g. up to 60 years of age at resection). Regardless of tumour test results, genetic testing can be indicated due to patient characteristics such as young age, personal or family cancer history.


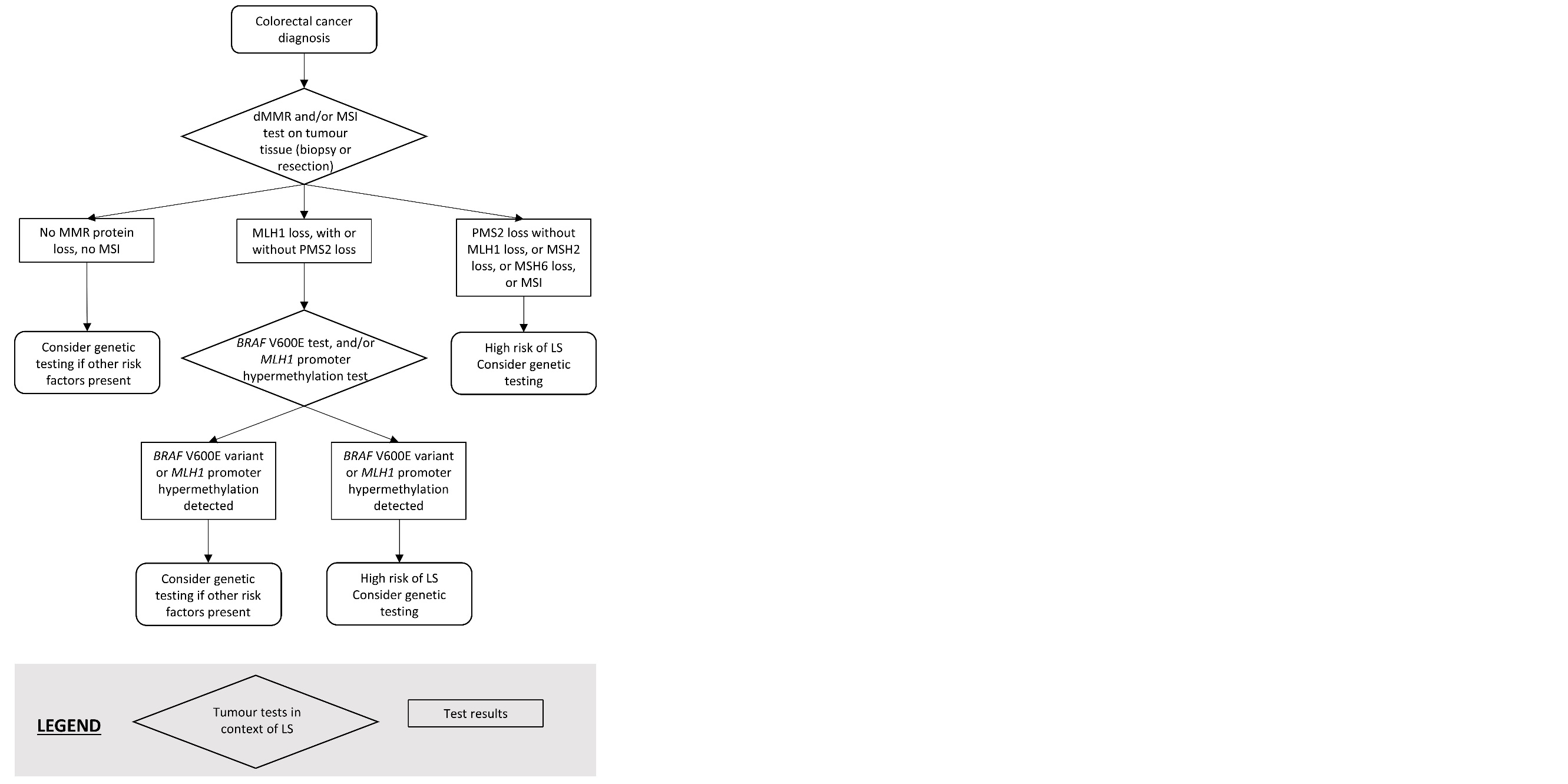

Supplement: Supplementary file 2 — Additional file 2: Conceptual map of a step-wise tumour testing approach for LS. [file 13053_2022_225_MOESM2_ESM.docx]
