# Supplementary material for: Lynch syndrome testing of colorectal cancer patients in a high-income country with universal healthcare: a retrospective study of current practice and gaps in seven australian hospitals
Source: Hered Cancer Clin Pract. 2022 May 4;20:18. doi: 10.1186/s13053-022-00225-1 (PMC9066828; doi:10.1186/s13053-022-00225-1)
Supplement: Supplementary file 3 — Additional file 3: Supplementary Methods. [file 13053_2022_225_MOESM3_ESM.docx]

**Lynch syndrome testing of colorectal cancer patients in a high-income country with universal healthcare: a retrospective study of current practice and gaps in seven Australian hospitals**

**SUPPLEMENTARY METHODS**

In most hospitals, patients were identified using diagnosis codes C18-C20 from the International Statistical Classification of Diseases and Related Health Problems, Tenth Revision, Australian Modification. CRC resections were identified using a consensus list of Medicare Benefits Schedule (MBS) codes, compiled in consultation with six clinicians (four surgeons, two gastroenterologists) at five hospitals and the internal study team (Additional File 7). The consensus list of MBS codes was also mapped to Australian Classification of Health Interventions (ACHI) codes and names of procedures in use at other hospitals (Additional File 8). For patients with multiple CRC resections in the study period, only data pertaining to the first resection were included.

***Hospital 1***

The hospital data analysis unit identified all patients who had a discharge date in the period 01/01/2017-31/01/2019, with a recorded diagnosis of colorectal cancer (ICD-10-AM).

Pathology data were extracted based on T/M codes (T codes: 6600, 6700, 6800 (appendix, colon, rectum), M codes: 8000-9999 (all cancer codes)) and pathology registration date for the period 01/07/2016-31/03/2019.

Patients with either surgery or a pathology record were initially included. These two datasets were then linked based on patient name, date of birth and MRN. For patients with missing surgery or pathology data in the initial extracts, the Implementation Lead manually extracted the missing information from electronic patient records. Following this, study inclusion/exclusion criteria were applied.

A query of the genetics services database was used to identify all individuals with colon cancer diagnosis in 2017-2018 who had a referral date in the period 01/12/2016-01/04/2019. The Implementation Lead linked the query results to the hospital data using patient name, date of birth and Medical Record Number (unique within the state’s public hospital system). In addition, a member of the genetics services team manually checked the database for names of all 11 patients at the hospital for whom the tumour test results indicated high risk of LS (verifying none of these patients had been missed in the query results).

***Hospital 2***

The hospital data unit identified all patients with a discharge date in the period 01/01/2017-31/12/2018, with a recorded diagnosis of colorectal cancer (ICD-10-AM) and a record of colorectal cancer resection (ACHI codes). Another hospital data unit then provided surgical variables using the MRNs from the initial data extraction.

The Implementation Lead manually extracted pathology data from histopathology reports on the hospital’s electronic records and linked this data based on patient name, date of birth and MRN.

To identify referrals to genetics services, data was linked to the genetics services centre attached to the hospital, using Medical Record Numbers.

***Hospital 3***

The hospital data unit identified all patients with a resection in the period 01/01/2017-31/12/2018, with a recorded diagnosis of colorectal cancer (ICD-10-AM) and a record of colorectal cancer resection (ACHI codes and MBS items).

The ACCORD database was used to obtain surgery data for these patients using patient name, Medical Record Number, date of birth, and ICD-10-AM, ACHI codes and MBS items.

The Implementation Lead manually extracted pathology data from the pathology database and linked this data based on Medical Record Number or patient name and date of birth.

To identify referrals to genetics services, data was linked to the genetics services centre attached to the hospital, using Medical Record Number, patient name and date of surgery.

To check no patients were missed, patient name, Medical Record Number and date of surgery were manually checked against a list of colorectal cancer patients maintained by the surgery department.

***Hospital 4***

The hospital data unit identified all patients whose hospital admission overlapped the period 01/01/2017-31/12/2018, with a recorded diagnosis of colorectal cancer (ICD-10-AM) and a record of colorectal cancer resection (ACHI codes mapped from MBS item codes, see Additional File 8). This data extract included pathology data.

To identify referrals to genetics services, we used a data query in the database used by the genetics services. This query included exact matches for the hospital site, with referral dates in the period 01/01/2017-28/02/2019, and patient age greater than 18 years. The Implementation Lead then restricted analysis using patient name, date of birth and Medical Record Number.

***Hospital 5***

The hospital data unit identified all patients whose hospital admission overlapped the period 01/01/2017-31/12/2018, with a recorded diagnosis of colorectal cancer (ICD-10-AM).

We linked the data to pathology records from the hospital pathology database based on patient name and date of birth.

To identify referrals to genetics services, we used a data query in the database used by the genetics services. This query included exact matches for the hospital site, with referral dates in the period 01/01/2017-28/02/2019, and patient age greater than 18 years. The Implementation Lead then restricted analysis using patient name, date of birth and Medical Record Number.

***Hospital 6***

The hospital data unit identified all patients with a resection in the period 01/01/2017-31/12/2018, with a recorded diagnosis of colorectal cancer (ICD-10-AM) and a record of colorectal cancer resection (ACHI codes).

The Implementation Lead manually extracted pathology data from histopathology reports on the hospital’s electronic records and linked these data based on patient name, date of birth and Medical Record Number.

To identify referrals to genetics services, we used a data query in the database used by the genetics services. This query included exact matches for the hospital site, with referral dates in the period 01/01/2017-28/02/2019, and patient age greater than 18 years. The Implementation Lead then restricted analysis using patient name, date of birth and Medical Record Number.

***Hospital 7***

The hospital data unit identified all patients whose hospital admission overlapped the period 01/01/2017-31/01/2019, with a recorded diagnosis of colorectal cancer (ICD-10-AM) and a record of colorectal cancer resection (ACHI codes).

The Implementation Lead manually extracted pathology data from the pathology database and linked this data based on Medical Record Number or patient name and date of birth.

To identify referrals to genetics services, we used a data query in the database used by genetics services. This query included exact matches for the hospital site, with referral dates in the period 01/01/2017-28/02/2019, and patient age greater than 18 years. The Implementation Lead then restricted analysis using patient name, date of birth and Medical Record Number.
