# Supplementary material for: Lynch syndrome testing of colorectal cancer patients in a high-income country with universal healthcare: a retrospective study of current practice and gaps in seven australian hospitals
Source: Hered Cancer Clin Pract. 2022 May 4;20:18. doi: 10.1186/s13053-022-00225-1 (PMC9066828; doi:10.1186/s13053-022-00225-1)
Supplement: Supplementary file 4 — Additional file 4: P-values for pairwise differences for missing test results and referrals to genetics services between hospitals. [file 13053_2022_225_MOESM4_ESM.docx]

**Lynch syndrome testing of colorectal cancer patients in a high-income country with universal healthcare: a retrospective study of current practice and gaps in seven Australian hospitals**

**P-values for pairwise differences for missing test results and referrals to genetics services between hospitals.**

The table shows p-values based on Fisher’s test, with significant differences (p<0.0024 to account for multiple testing, see Methods) highlighted in bold with yellow background.

| **dMMR/MSI test results** | | | | | | | |
| --- | --- | --- | --- | --- | --- | --- | --- |
| Hospital  [% missing] | H1 [37%] | H2 [8%] | H3 [11%] | H4 [14%] | H5 [9%] | H6 [2%] | H7 [0%] |
| H1 [37%] |  | **9.4E-21** | **2.0E-08** | **6.2E-11** | **4.4E-15** | **1.8E-21** | **2.1E-19** |
| H2 [8%] |  |  | 3.5E-01 | 8.5E-03 | 6.5E-01 | 5.3E-03 | **4.8E-04** |
| H3 [11%] |  |  |  | 4.3E-01 | 5.8E-01 | **1.3E-03** | **1.9E-04** |
| H4 [14%] |  |  |  |  | 6.5E-02 | **3.0E-06** | **7.5E-07** |
| H5 [9%] |  |  |  |  |  | 2.6E-03 | **2.3E-04** |
| H6 [2%] |  |  |  |  |  |  | 2.7E-01 |
| ***BRAF* V600E and *MLH1* promoter hypermethylation (among patients with MLH1 loss only)** | | | | | | | |
| Hospital  [% missing] | H1 [0%] | H2 [28%] | H3 [9%] | H4 [7%] | H5 [59%] | H6 [8%] | H7 [0%] |
| H1 [0%] |  | 6.1E-03 | 3.4E-01 | 5.0E-01 | **3.0E-06** | 4.9E-01 | 1.0E+00 |
| H2 [28%] |  |  | 2.6E-01 | 3.9E-02 | 5.2E-03 | 6.7E-02 | 2.6E-02 |
| H3 [9%] |  |  |  | 1.0E+00 | 5.2E-03 | 1.0E+00 | 4.2E-01 |
| H4 [7%] |  |  |  |  | **2.5E-05** | 1.0E+00 | 5.3E-01 |
| H5 [59%] |  |  |  |  |  | **2.5E-05** | **3.9E-05** |
| H6 [8%] |  |  |  |  |  |  | 5.2E-01 |
| **Referral to genetics services (among patients with tumour tests completed and indicating high LS risk only)** | | | | | | | |
| Hospital  [% missing] | H1 [45%] | H2 [50%] | H3 [25%] | H4 [29%] | H5 [60%] | H6 [67%] | H7 [50%] |
| H1 [45%] |  | 1.0E+00 | 6.0E-01 | 4.3E-01 | 6.7E-01 | 4.3E-01 | 1.0E+00 |
| H2 [50%] |  |  | 5.8E-01 | 4.2E-01 | 6.9E-01 | 4.5E-01 | 1.0E+00 |
| H3 [25%] |  |  |  | 1.0E+00 | 5.6E-01 | 2.6E-01 | 5.8E-01 |
| H4 [29%] |  |  |  |  | 2.1E-01 | 6.6E-02 | 3.9E-01 |
| H5 [60%] |  |  |  |  |  | 1.0E+00 | 1.0E+00 |
| H6 [67%] |  |  |  |  |  |  | 6.6E-01 |
