# Supplementary material for: Lynch syndrome testing of colorectal cancer patients in a high-income country with universal healthcare: a retrospective study of current practice and gaps in seven australian hospitals
Source: Hered Cancer Clin Pract. 2022 May 4;20:18. doi: 10.1186/s13053-022-00225-1 (PMC9066828; doi:10.1186/s13053-022-00225-1)
Supplement: Supplementary file 5 — Additional file 5: Diagnostic genetic testing among patients with complete, high-risk tumour test results who had a referral to genetic services. [file 13053_2022_225_MOESM5_ESM.docx]

**Lynch syndrome testing of colorectal cancer patients in a high-income country with universal healthcare: a retrospective study of current practice and gaps in seven Australian hospitals**

**Diagnostic genetic testing among patients with complete, high-risk tumour test results who had a referral to genetic services.**

2 patients were referred to other genetics services for which attendance records were unavailable; the numbers of patients who attended genetics services appointments and those who has a diagnostic genetic test included some patients for whom the referral and/or testing was carried out prior to resection (e.g. due to younger age, a previous cancer diagnosis, or family history).
P/LP: pathogenic/likely pathogenic variant; VUS: variant of unknown significance.

**
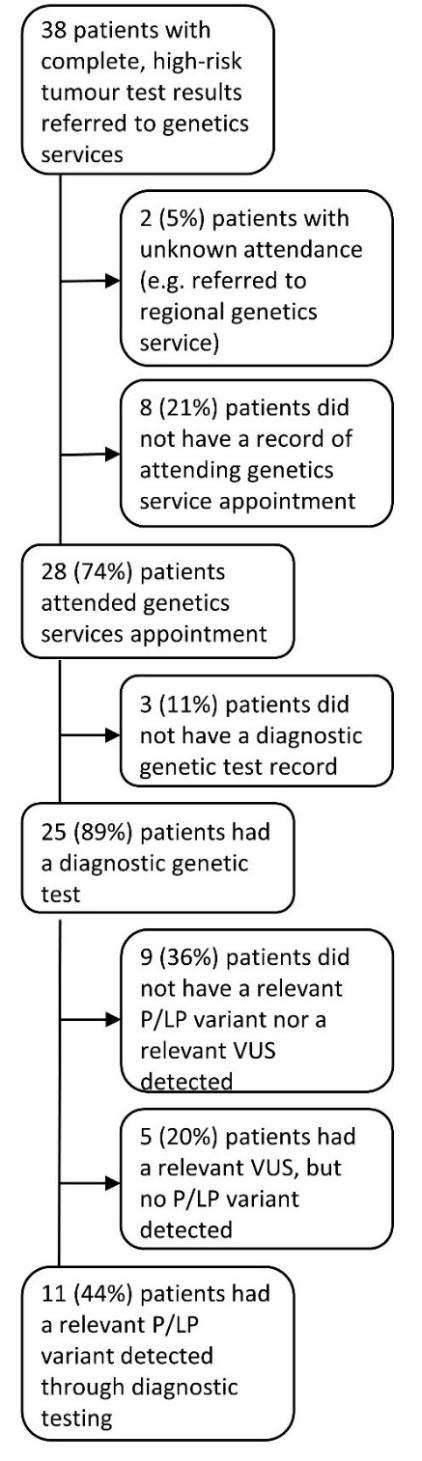
**
