# Supplementary material for: Lynch syndrome testing of colorectal cancer patients in a high-income country with universal healthcare: a retrospective study of current practice and gaps in seven australian hospitals
Source: Hered Cancer Clin Pract. 2022 May 4;20:18. doi: 10.1186/s13053-022-00225-1 (PMC9066828; doi:10.1186/s13053-022-00225-1)
Supplement: Supplementary file 6 — Additional file 6: Examples of different terminology for LS tumour test results used in pathology reports. [file 13053_2022_225_MOESM6_ESM.docx]

**Lynch syndrome testing of colorectal cancer patients in a high-income country with universal healthcare: a retrospective study of current practice and gaps in seven Australian hospitals**

**Examples of different terminology for LS tumour test results used in pathology reports.**

| Hospital | Results that indicate high risk of LS | Results that do not indicate high risk of LS |
| --- | --- | --- |
| H1 | Example 1: "It is noted that there was loss of expression of the DNA mismatch repair proteins MLH1 and PMS2 by the tumour"  Example 2: "Immunohistochemistry shows retained expression of MLH1, MSH2 and MSH6 in the tumour nuclei. However there is loss of PMS2 staining. This raises the possibility of a germline PMS2 mutation (Lynch syndrome)."  Example 3: "There is loss of PMS2 protein expression by the tumour with retained MLH1, MSH2 and MSH2; the findings suggest a microsatellite unstable status. Negative staining with BRAF-VE1 suggests the presence of BRAFV600E mutation is unlikely. A Lynch syndrome related carcinoma is a potential consideration and correlation with additional molecular testing including MSI status, clinical setting and family history is recommended, with consideration given to referral to Genetic Services WA as appropriate." | Example 1: "There is no loss of expression of the mismatch repair proteins MLH1, PMS2, MSH2, and MSH6."  Example 2: "There is no staining loss for MLH1, MSH2, MSH6 and PMS2."  Example 3: " There is no loss of MLH1, MSH2, MSH6 and PMS2 protein expression by the tumour; 'HNPCC' would be unlikely in these circumstances, but the results should be interpreted in conjunction with the clinical setting and family history. MSI could be performed if requested. Comment - It should be noted that testing with immunohistochemistry alone may miss a small fraction of HNPCC cases, as there may be mutations in other mismatch repair genes not currently tested for. If there is high clinical suspicion of HNPCC, further advice should be sought from Genetic Services."  Example 4: "With the above MSI profile, Lynch Syndrome related tumour i.e. 'HNPCC' would be unlikely but the results should be interpreted in conjunction with the clinical setting, and family history. If there is a high clinical suspicion of HNPCC, further advice should be sought from Genetic Services WA." |
| H2 | *[examples not available]* | *[examples not available]* |
| H3 | Example 1: "While staining patterns of MLH1 and PMS2 are unusual, they are abnormal and suggest that the tumour is mismatch repair deficient (MSI)…maybe associated with Lynch syndrome or sporadic inactivation of MLH1" and "In this setting the absence of a BRAF mutation indicates the tumour may have been caused by Lynch syndrome. Referral to FCC is indicated."  Example 2: "..high probability HNPCC/Lynch syndrome. Recommend referral to FCC"  Example 3: "Mismatch repair deficient (dMMR). Pattern associated with Lynch syndrome and referral to the FCC is indicated." Example 4: "dMMR, MSI-instability, MSI+"  Example 5: "R/T FCC - Lynch syndrome" | Example 1: "(pMMR)"  Example 2: "unlikely Lynch syndrome"  Example 3: "MLH1 and PMS2 are not expressed. MSH2 and MSH6 are normally expressed. BRAF is positive." |
| H4 | *[examples not available]* | *[examples not available]* |
| H5 | Example 1: "MLH1 - Loss of expression PMS2 - Loss of expression MSH2 - No loss of expression MSH6 - No loss of expression"  Example 2: "MLH1 staining: negative MSH2 staining: positive MSH6 staining: positive PMS2 staining: negative There is loss of MLH1 and PMS2 mismatch repair gene expression"  Example 3: "Loss of expression for MLH1 and PMS2 present. Many tumours with this immunohistochemical phenotype occur as a result of epigenetic silencing; however, a proportion will be as a result of a germline mutation. Immunohistochemistry for BRAF mutation may be undertaken if required"  Example 4: "Loss of expression for MLH1 and PMS2 present, this can be epigenetic silencing or germline mutation; genetic counselling and further testing for BRAF mutation and/ or Microsatellite instability should be considered." | Example 1: "Normal expression pattern NO LOSS OF EXPRESSION OF MSI-IHC MARKERS." |
| H6 | Example 1: "This is an abnormal pattern of staining indicating microsatellite instability phenotype (MSI-H). In lieu of loss of staining for PMS2 and MLH1, and negative BRAF staining, this may reflect a germline mutation (by hypermethylation) of the MLH1 mismatch repair gene. However immunohistochemistry does not always correlate with the molecular testing, hence correlation with clinical and family history is suggested, and further investigations are recommended."  Example 2: "Immunoperoxidase stains for the mismatch repair enzymes MLH1, PMS2 , MSH2 and MSH6 show loss of staining of carcinoma nuclei for MLH1 and its binding partner PMS2. MLH1-deficient colorectal carcinoma can be caused by Lynch syndrome or by sporadic MLH1 methylation. In patients over 70 years of age, sporadic MSI cancer is usual. A B-RAF mutation is supportive of sporadic MLH1 methylation. However, as BRAF staining is negative in the current case, Lynch syndrome is a possibility. Clinical correlation is recommended."  Example 3: "This is an abnormal pattern of staining indicating loss of expression of the MLH1 gene. High level microsatellite instability is likely, which may be sporadic of familial (Lynch syndrome). BRAF IHC is in progress. Please correlate with patient's personal and family cancer history." and "This suggests an absence of BRAF V600E mutation and suggests that the loss of expression of MLH1 may be familial, ie. Lynch syndrome, rather than sporadic. Correlation with the patient's personal and family cancer history is suggested." | Example 1: "This is a normal pattern of staining. High level microsatellite instability (MSI-H) is unlikely."  Example 2: "This is a normal pattern of staining, hence MSI-H is unlikely. Please correlate with the clinical findings."  Example 3: "As the BRAF V600E immunostain has a very high sensitivity and specificity (both close to 100%) for the BRAF V600E mutation, its positive staining virtually confirms an underlying BRAF V600E mutation. This is associated with sporadic colorectal cancers due to MLH1 promoter methylation. As such, Lynch syndrome is unlikely."  Example 4: "Immunoperoxidase stains for the mismatch repair enzymes MLH1, PMS2 , MSH2 and MSH6 show loss of staining of carcinoma nuclei for MLH1 and its binding partner PMS2. MLH1-deficient colorectal carcinoma can be caused by Lynch syndrome or by sporadic MLH1 methylation. In patients over 70 years of age, sporadic MSI cancer is usual. A B-RAF mutation is supportive of sporadic MLH1 methylation." |
| H7 | Example 1: "The negative staining for PMS2 and MLH1 indicates a microsatellite unstable phenotype (due to inactivation of MLH1 or PMS2). Two thirds of colorectal carcinomas which show silencing of MLH1/PMS2 due to somatic hypermethylation are associated with BRAFV600E mutation whereas less than 1% of colorectal carcinomas associated with Lynch syndrome show BRAFV600E mutation. Therefore the possibility of Lynch syndrome should be considered clinically."  Example 2: "The negative staining for PMS2 indicates a microsatellite unstable phenotype. This could be caused by MLH1 promoter hypermethylation or Lynch syndrome"  Example 3: "Negative staining for MSH6 is highly correlated with Lynch syndrome due to germline mutation of MSH6. Therefore referral for formal genetic counselling and testing is recommended" | Example 1: "The positive staining for PMS2, MLH1, MSH2 and MSH6 indicates a microsatellite stable phenotype"  Example 2: "The negative staining for PMS2 and MLH1 indicates a microsatellite unstable phenotype. Less than 1% of Lynch syndrome colorectal carcinomas are associated with BRAFV600E mutation, therefore the presence of positive staining for BRAFV600E virtually excludes Lynch syndrome despite the presence of microsatellite instability." |
