# Supplementary material for: Lynch syndrome testing of colorectal cancer patients in a high-income country with universal healthcare: a retrospective study of current practice and gaps in seven australian hospitals
Source: Hered Cancer Clin Pract. 2022 May 4;20:18. doi: 10.1186/s13053-022-00225-1 (PMC9066828; doi:10.1186/s13053-022-00225-1)
Supplement: Supplementary file 7 — Additional file 7: Consensus list of Medicare Benefit Schedule (MBS) procedure codes used to identify colorectal resections (in conjunction with ICD codes for CRC diagnosis). [file 13053_2022_225_MOESM7_ESM.docx]

**Lynch syndrome testing of colorectal cancer patients in a high-income country with universal healthcare: a retrospective study of current practice and gaps in seven Australian hospitals**

#### **Consensus list of Medicare Benefit Schedule (MBS) procedure codes used to identify colorectal resections (in conjunction with ICD codes for CRC diagnosis).**

| **MBS item** | **Description** |
| --- | --- |
| 32000 | LARGE INTESTINE, resection of, without anastomosis, including right hemicolectomy (including formation of stoma) |
| 32003 | LARGE INTESTINE, resection of, with anastomosis, including right hemicolectomy |
| 32004 | LARGE INTESTINE, subtotal colectomy (resection of right colon, transverse colon and splenic flexure) without anastomosis, not being a service associated with a service to which item 32000, 32003, 32005 or 32006 applies |
| 32005 | LARGE INTESTINE, subtotal colectomy (resection of right colon, transverse colon and splenic flexure) with anastomosis, not being a service associated with a service to which item 32000, 32003, 32004 or 32006 applies |
| 32006 | LEFT HEMICOLECTOMY, including the descending and sigmoid colon (including formation of stoma) |
| 32009 | TOTAL COLECTOMY AND ILEOSTOMY |
| 32012 | TOTAL COLECTOMY AND ILEORECTAL ANASTOMOSIS |
| 32015 | TOTAL COLECTOMY WITH EXCISION OF RECTUM AND ILEOSTOMY  1 surgeon |
| 32018 | TOTAL COLECTOMY WITH EXCISION OF RECTUM AND ILEOSTOMY, COMBINED SYNCHRONOUS OPERATION; ABDOMINAL RESECTION |
| 32021 | TOTAL COLECTOMY WITH EXCISION OF RECTUM AND ILEOSTOMY, COMBINED SYNCHRONOUS OPERATION; PERINEAL RESECTION |
| 32024 | RECTUM, HIGH RESTORATIVE ANTERIOR RESECTION WITH INTRAPERITONEAL ANASTOMOSIS (of the rectum) greater than 10 centimetres from the anal verge  excluding resection of sigmoid colon alone not being a service associated with a service to which item 32103, 32104 or 32106 applies |
| 32025 | RECTUM, LOW RESTORATIVE ANTERIOR RESECTION WITH EXTRAPERITONEAL ANASTOMOSIS (of the rectum) less than 10 centimetres from the anal verge, with or without covering stoma not being a service associated with a service to which item 32103, 32104 or 32106 applies |
| 32026 | RECTUM, ULTRA LOW RESTORATIVE RESECTION, with or without covering stoma, where the anastomosis is sited in the anorectal region and is 6cm or less from the anal verge |
| 32028 | RECTUM, LOW OR ULTRA LOW RESTORATIVE RESECTION, with peranal sutured coloanal anastomosis, with or without covering stoma |
| 32030 | RECTOSIGMOIDECTOMY  (Hartmann's operation) |
| 32036 | SACROCOCCYGEAL AND PRESACRAL TUMOUR  excision of |
| 32039 | RECTUM AND ANUS, ABDOMINOPERINEAL RESECTION OF  1 surgeon |
| 32042 | RECTUM AND ANUS, ABDOMINOPERINEAL RESECTION OF, COMBINED SYNCHRONOUS OPERATION  abdominal resection |
| 32045 | RECTUM AND ANUS, ABDOMINOPERINEAL RESECTION OF, COMBINED SYNCHRONOUS OPERATION  perineal resection |
| 32046 | RECTUM and ANUS, abdomino-perineal resection of, combined synchronous operation - perineal resection where the perineal surgeon also provides assistance to the abdominal surgeon |
| 32051 | TOTAL COLECTOMY with excision of rectum and ileoanal anastomosis with formation of ileal reservoir, with or without creation of temporary ileostomy  1 surgeon |
| 32054 | TOTAL COLECTOMY with excision of rectum and ileoanal anastomosis with formation of ileal reservoir, with or without creation of temporary ileostomy  conjoint surgery, abdominal surgeon (including aftercare) |
| 32057 | TOTAL COLECTOMY with excision of rectum and ileoanal anastomosis with formation of ileal reservoir  conjoint surgery, perineal surgeon |
| 32099 | RECTAL TUMOUR of 5 centimetres or less in diameter, per anal submucosal excision of |
| 32102 | RECTAL TUMOUR of greater than 5 centimetres in diameter, indicated by pathological examination, per anal submucosal excision of |
| 32103 | RECTAL TUMOUR, of less than 4 cm in diameter, per anal excision of, using rectoscopy incorporating either 3 dimensional or 2 dimensional optic viewing systems, if removal is unable to be performed during colonoscopy or by local excision, other than a service associated with a service to which item 32024, 32025, 32104 or 32106 applies |
| 32104 | RECTAL TUMOUR, of 4 cm or greater in diameter, per anal excision of, using rectoscopy incorporating either 3 dimensional or 2 dimensional optic viewing systems, if removal is unable to be performed during colonoscopy or by local excision, other than a service associated with a service to which item 32024, 32025, 32103 or 32106 applies |
| 32105 | ANORECTAL CARCINOMA  per anal full thickness excision of |
| 32106 | ANTEROLATERAL INTRAPERITONEAL RECTAL TUMOUR, per anal excision of, using rectoscopy incorporating either 3 dimensional or 2 dimensional optic viewing systems, if removal is unable to be performed during colonoscopy and if removal requires dissection within the peritoneal cavity, other than a service associated with a service to which item 32024, 32025, 32103 or 32104 applies |
| 32108 | RECTAL TUMOUR, transsphincteric excision of (Kraske or similar operation) |
